# Interventions for Prevention of Tobacco Smoking in School-Aged Children and Adolescents: A Systematic Review and Meta-Analysis

**DOI:** 10.7759/cureus.77008

**Published:** 2025-01-06

**Authors:** Fahad A Alsahli, Naif M Alruwais, Leyan S Alsultan, Bander S Abojalid, Reem O Nughays, Abdulsalam M Humedi, Abdulaziz Alosaimi, Mohamed T Alrubaian, Dhai Z Almuteri, Muath A Alkhunizan

**Affiliations:** 1 College of Medicine, Shaqra University, Riyadh, SAU; 2 College of Medicine, Imam Abdulrahman Bin Faisal University, Dammam, SAU; 3 College of Medicine, King Saud Bin Abdulaziz University for Health Sciences, Riyadh, SAU; 4 College of Medicine, Jazan University, Jazan, SAU; 5 College of Medicine, Alfaisal University, Riyadh, SAU; 6 General Practice, Unaizah College of Medicine and Medical Sciences, Qassim University, Unaizah, SAU; 7 Family Medicine, King Faisal Specialist Hospital and Research Centre, Riyadh, SAU

**Keywords:** adolescents, behavior-based interventions, non-pharmacological interventions, smoking cessation, tobacco prevention

## Abstract

Smoking is a major worldwide health concern and a leading cause of preventable diseases such as cancer and heart disease. Adolescence, marked by experimentation and risk-taking behaviors, is a critical developmental stage where tobacco smoking frequently begins. Early smoking is associated with an increased risk of health problems, reduced life expectancy, and lifetime addiction, making prevention during this stage imperative. Despite its urgency, evidence on effective non-pharmacological preventative techniques for this demographic remains limited. This systematic review and meta-analysis aimed to assess the components, efficacy, and potential negative consequences of behavior-based, non-pharmacological interventions. A systematic search of PubMed and the Cochrane Library was conducted using Preferred Reporting Items for Systematic Reviews and Meta-Analyses (PRISMA) criteria for randomized controlled trials (RCTs) published between 2014 and 2024. Eligible studies included school-aged children and evaluated smoking initiation or cessation as outcomes. Data from six RCTs involving 10,192 participants were analyzed using Review Manager (RevMan v5.4, The Cochrane Collaboration, Oxford, UK). Risk ratios (RR) with 95% confidence intervals (CI) and I² statistics were calculated to evaluate heterogeneity and intervention efficacy. Results showed that school-based educational programs significantly reduced smoking initiation rates at six months (RR = 0.38, 95% CI: 0.23-0.61, p < 0.001), though effects diminished at longer follow-up periods (12-36 months). Culturally tailored, peer-led interventions demonstrated moderate efficacy in improving attitudes toward smoking and reducing consumption. Combined interventions were the most effective overall, but variability in study design and follow-up durations limited generalizability. This research highlights the short-term effectiveness of school-based and culturally sensitive interventions in reducing adolescent tobacco use. Future research should prioritize long-term strategies that integrate digital tools, family, and community involvement to sustain behavioral changes and combat the global tobacco epidemic effectively.

## Introduction and background

Worldwide, tobacco use is a major contributor to avoidable illnesses, such as coronary heart disease and certain forms of cancer [1,2]. Despite international public health initiatives, smoking is still a major problem, especially among teenagers, whose adolescence is frequently marked by experimentation and risk-taking, including tobacco use. For instance, almost 25% of American high school students and 8% of middle school students reported smoking tobacco in 2014; of these, 9.2% and 2.5%, respectively, were current smokers [3,4].

The fact that teen smoking frequently continues into adulthood makes it a serious public health concern [5]. Those who start smoking before reaching maturity are more likely to develop nicotine dependence, have a lesser chance of stopping, and are more vulnerable to mental and physical health problems [6,7]. Additionally, studies show that smokers are less likely than nonsmokers to live for at least 10 years [3-9].

Since the majority of tobacco users start smoking during this developmental stage, preventing tobacco use throughout adolescence is essential to combating the global tobacco epidemic [10]. Adolescent smoking incidence is rising in Arab nations, with notable regional variations, making this issue particularly urgent [11,12]. In order to reduce the increasing burden of tobacco use in this susceptible group, these concerning developments highlight the urgent need for focused, efficient prevention initiatives [13,14].

This study aimed to conduct an up-to-date systematic review and meta-analysis of trials to evaluate the effectiveness of non-pharmacological, behavior-based therapies in reducing tobacco use among school-aged children, identify the elements of effective preventative measures, and assess any potential negative consequences.

## Review

Methods

This systematic review was done to assess the efficacy of non-pharmacological, behavior-based therapies in preventing tobacco use among children and adolescents [3-11,15]. The review followed Preferred Reporting Items for Systematic Reviews and Meta-Analyses (PRISMA) standards [12] to ensure accuracy and transparency, and the protocol was registered in PROSPERO (Registration ID: CRD42024588571).

Search Strategy

The Cochrane Library and PubMed were thoroughly searched for research published between 2014 and 2024 [16]. Using Boolean operators, the search terms included keywords like "primary care," "prevent," "tobacco use," "smoking," "adolescents," and their synonyms.

PubMed Keywords

("primary care" OR "healthcare provider") AND (prevent OR stop OR reduce OR avoid) AND ("tobacco use" OR smoking OR nicotine OR vaping) AND (children OR adolescents OR teenagers OR youth).

Cochrane Library Keywords

#1 ("primary care" OR "healthcare provider" OR clinician OR pediatrician OR "family physician") #2 (prevent OR stop OR reduce OR avoid) #3 ("tobacco use" OR smoking OR "cigarette use" OR nicotine OR vaping) #4 (children OR adolescents OR teenagers OR youth OR minors) #5 #1 AND #2 AND #3 AND #4

Rayyan AI (Rayyan Systems, Inc., Cambridge, Massachusetts, USA) was used to find and eliminate duplicate records, leaving 1,341 distinct studies for screening [16]. In order to find any pertinent articles, the reference lists of the included research were also manually examined.

Eligibility Criteria

Inclusion and exclusion criteria were defined using the PICOTS framework (Population, Intervention, Comparator, Outcomes, Timing, and Setting). The population included children and teenagers in school, aged from 6 to 18. The intervention consisted of non-pharmacological, behavior-based interventions aimed at preventing tobacco initiation. The comparator was either no intervention or standard treatment. The primary outcomes were the rates of smoking initiation and cessation, while secondary outcomes included changes in attitudes, smoking intentions, or smoking behaviors. The timing included research with any duration of follow-up, and the context was community-based programs or primary care.

Non-randomized trials, observational studies, and publications that were not available in English were among the studies that were excluded. These exclusions were intended to maintain consistency in scientific rigour and data interpretation.

Study Selection

To ensure accuracy and efficiency, two independent reviewers used Rayyan AI to screen all records' titles and abstracts [16]. After that, separate full-text reviews were carried out for the papers that qualified, and a third reviewer settled any disputes. Results include the PRISMA flow diagram that summarizes this procedure [12].

Data Extraction

A standardized data extraction form was developed and pilot-tested to ensure consistency [17]. Information extracted from each study included study characteristics (author, year of publication, country, study design, sample size, and follow-up duration), participant characteristics (age, sex, smoking history, and exposure to secondhand smoke), intervention details (type, duration, frequency, and delivery method, such as school-based or peer-led), and outcomes. Primary outcomes focused on smoking initiation and cessation rates, while secondary outcomes included factors like attitudes toward smoking and quality of life. Data extraction was conducted by two independent reviewers, with discrepancies resolved through discussion [18]. Missing data were addressed by contacting study authors when possible [19].

Risk of Bias Assessment

Two reviewers independently assessed the risk of bias using the Revised Cochrane Risk of Bias Tool (RoB 2) [20]. Five major areas. were evaluated by this tool: outcome measurement, missing data, randomization, variations from planned interventions, and selective reporting. Excluded from the meta-analysis were studies that had a high risk of bias.

Statistical Analysis

Review Manager (RevMan v5.4, The Cochrane Collaboration, Oxford, UK) was used to conduct meta-analyses [21]. Mean differences (MD) were used to investigate continuous variables (like improvements in quality of life), while risk ratios (RR) with 95% confidence interval (CI) were used to assess categorical outcomes (like smoking initiation). Heterogeneity was measured using the I² statistic, with thresholds for low (<30%), moderate (30-60%), and high (>60%) heterogeneity [6,7]. Subgroup and sensitivity analyses were designed to address heterogeneity and evaluate the findings' validity [15]. A p-value of less than 0.05 was considered statistically significant. This systematic review uses rigorous, standardized procedures to evaluate the efficacy of programs aimed at preventing teenage smoking. Transparency, repeatability, and excellent scientific quality are guaranteed by the study's adherence to PRISMA criteria [12] and the use of sophisticated screening techniques such as Rayyan AI [16].

Results

PRISMA Diagram

**Figure 1:**
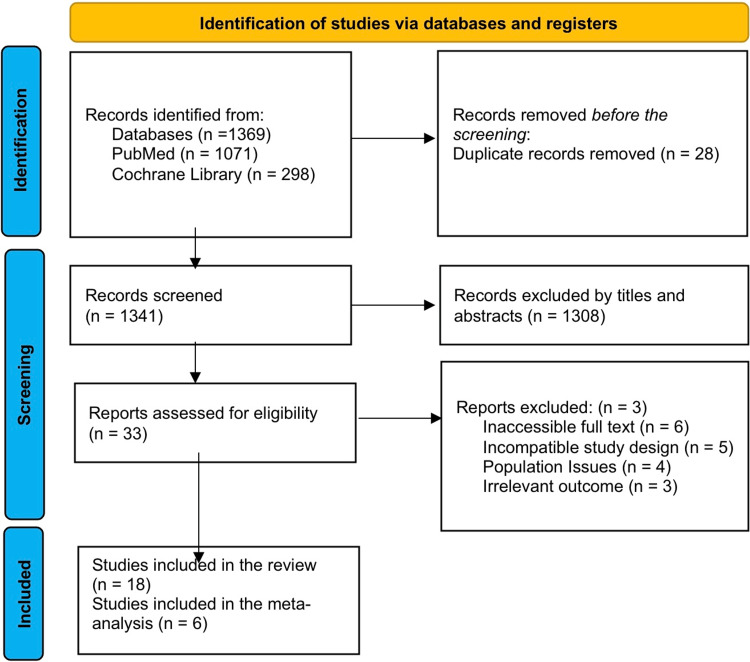
Flow diagram of study selection for the systematic review Reference: [12]

A total of 1369 publications were collected from two database searches (PubMed and Cochrane). Of them, 28 were left out because they were duplicates. Six papers were selected for the meta-analysis (Figure 1) [12].

Overview of the Included Studies

The research involved 10,192 participants from schools and primary care clinics, with ages ranging from 9 to 17 years. These studies were carried out between 2014 and 2018 in a number of nations, including the United States, the Netherlands, Spain, and Saudi Arabia [3,6,11,13,17,19]. Randomized controlled trials (RCTs) from one month to three years comprised all of the studies listed in the table. These studies' interventions included a range of smoking prevention tactics for teenagers, such as mail-delivered activity modules, web-based programs with customized messages, short counseling sessions with follow-up calls, peer-led programs that were culturally appropriate, persuasive messages to encourage resource engagement, and a multi-lesson, long-term comprehensive education program (Table 1).

**Table 1:** The baseline characteristics of included studies in the meta-analysis RCT: randomized controlled trial; I: intervention; C: control

First author, year	Study design/setting	No. of experimental patients/control	Follow-up duration	The mean (SD) age of the participants	Gender	Race	Smoking risk	Intervention
Hiemstra, 2014 [6]	418 elementary schools in the Netherlands RCT study	N=1478 (intervention: 728 vs. control: 750)	3 years (36 months)	Total: 10.10 ± 0.78; Intervention: 10.13 ± 0.78; Control:10.08 ± 0.77; Range: 9-11	Total: 663 males, 735 females; Intervention: 297 males, 387 females; Control: 366 males, 348 females	Total: 1372 Dutch, 25 others; Intervention: 675 Dutch, 9 others; Control: 697 Dutch, 16 others	Smoking status parents: Both never smokers: 317 (150 I, 167 C); One former and one never smoker: 333 (173 I, 160 C); Both former smokers: 229 (112 I, 117 C); One current and one never smoker: 176 (83 I, 93 C); One current and one former smoker: 159 (78 I, 81 C); Both current smokers:158 (77 I, 81C)	Intervention: Five activity modules, including a communication sheet for mothers, were received by mail at four-week intervals, along with one booster module one year after the baseline. Control: received a fact-based intervention only.
Cremers, 2015 [3]	162 schools in the Netherlands RCT study	N=3213 (intervention: (Prompt: 1207, No-prompt:1003) vs. control: 1003)	2 years (25 months)	Total: 10.36 ± 0.55; Intervention: Prompt:10.36 ± 0.55, No prompt: 10.35 ± 0.54, Control: 10.38 ± 0.55; Range: 10-12	Total: 1588 males, 1625 females; Intervention: Prompt: 589 males, 618 females; No-prompt: 508 males, 495 females; Control:512 males, 491 females	NA	NA	Intervention: Web-based, computer-tailored; Results programs supplemented with prompt messages; Control: None; Under supervision by their teachers
Pbert, 2015 [17]	8 pediatric Primary care clinics in central Massachusetts, USA RCT study	N=2711	1 year (12 months)	Range: 13-17	NA	NA	NA	Intervention: brief counseling by pediatric providers, one in-person visit, and four follow-up calls from peer counselors aged 21 to 25, all based on the U.S. Public Health Service's 5A model. Control: Usual care
Mohammed, 2016 [13]	Secondary school in Taif, Saudia Arabia RCT	N=1416 (Intervention: 709 vs. control: 707)	6 months	Total: 13.88 ± 0.60; Intervention: 13.90 ± 0.61; Control:13.86 ± 0.70; Range: 13-15	Males only	NA	NA	Intervention: The smoking prevention program was translated and adapted to fit Saudi local culture and norms, followed by peer-led group work and active learning. Control: Usual curriculum 5 lessons (45 min each)
Mays, 2017 [19]	Adolescent medicine clinic in a large, urban hospital in the USA RCT	N=319	1 month	Total: 15.0 ± 1.6; Range: 12-17	Males: 105, Females: 204	Black/African American: 84, White: 184, Other: 51	Never smoker: 145; Susceptible/ever smoker: 169; Exposure to family members’ smoking: 59; Exposure to friends’ smoking: 85	Intervention: persuasive gain and loss-framed messages for motivating adolescents to engage with an evidence-based smoking prevention website. Control: Neutral message
Leiva, 2018 [11]	22 secondary schools in Spain RCT	N=1055 (Intervention: 466 vs. control: 590)	3 years (36 months)	Intervention: 12.3 ± 0.71; Control: 12.2 ± 0.64; Range: 13-15	Intervention: 513 males, 507 females; Control: 681 males, 715 females	NA	Smoking status: Daily smoking: 14 I, 11 C; Weekly smoking: 6 I, 16 C; Occasional smoking: 123 I, 158 C; Never smoker: 864 I, 1196 C; Quit smoker: 7 I, 9 C; Exposure to family members’ smoking: Mother and father: 246 I, 265 C; Only father: 165 I, 240 C; Only mother: 159 I, 188 C; Sibling smoking: 148 I, 170 C	Intervention: ITACA smoking prevention education program (4-year curricular component consisting of 22 lessons) Control: None

The smoking results following the therapies are summarized in Table 2. While some studies found no substantial impact on smoking behaviors or start, others found changes in attitudes and levels of engagement. For more successful smoking prevention, common recommendations stressed the significance of focusing on older adolescents, integrating interventions across communities, families, and schools, and taking peer leaders and life skills training into consideration.

**Table 2:** Outcomes of the included studies in the meta-analysis I: intervention; C: control

First author, year	Abstinence rates	Smoking initiation	Smoking intention/attitude	Smoking consumption	Conclusion and recommendations
Hiemstra, 2014 [6]	Intervention: 610/684; Control: 628/714	6-month follow-up: (I:5/670, C: 13/735); 12-month follow-up: (I:10/646, C: 18/713); 24-month follow-up: (I:24/633, C: 37/694); 36-month follow-up: (I:63/616, C: 79/689)	NA	NA	No effects on smoking initiation were found after 36 months. It is possible that the program was implemented with children who were too young. Future programs should be tested with participants closer to the age of smoking onset.
Cremers, 2015 [3]	NA	12-month follow-up: (I:6/1376, C: 3/718); 24-month follow-up: (I:8/974, C: 5/488)	12-month follow-up: (I:31/1324, C: 17/682); 24-month follow-up: (I:17/937, C: 6/465)	NA	This study found that web-based, computer-tailored feedback - both with and without prompts - did not effectively change children's smoking intentions or behaviors compared to no information. Future prevention programs should target children closer to the age of smoking onset and focus on managing exposure to educational content and responses to prompts.
Pbert, 2015 [17]	NA	NA	NA	NA	The primary outcome was self-reported smoking abstinence in the past 30 days, which is a direct measure of the intervention's effectiveness and an important outcome for patients.
Mohammed, 2016 [13]	Intervention: 528/698; Control: 519/683	6-month follow-up: (I:17/528, C: 46/519); P=0.019	Intervention: More negative attitude (Mean Δ = -0.15, p < 0.01); Control: Neutral/positive attitude (Mean Δ = 0.01)	Intervention: 145/698; Control: 202/683	Post-intervention, respondents in the experimental group expressed a more negative attitude toward smoking, stronger anti-smoking norms, higher confidence in staying non-smokers, increased action planning to avoid smoking, and lower future smoking intentions compared to the control group.
Mays, 2017 [19]	NA	NA	NA	NA	The study showed that engagement was significantly higher with the loss-framed message than with the gain-framed or neutral messages. Future research should use objective website engagement measures and examine smoking behavior as an outcome.
Leiva, 2018 [11]	Intervention: 453/465; Control: 573/588	36-month follow-up: Intervention: 112/465; Control: 128/588	NA	Intervention: 13/465; Control: 15/588	The results show that this program had no significant impact on adolescent smoking rates. More effective strategies might include integrated interventions involving schools, families, and community efforts. Other options to explore include targeting high-risk groups, using peer leaders for interventions, and combining life skills training with community initiatives.

**Figure 2:**
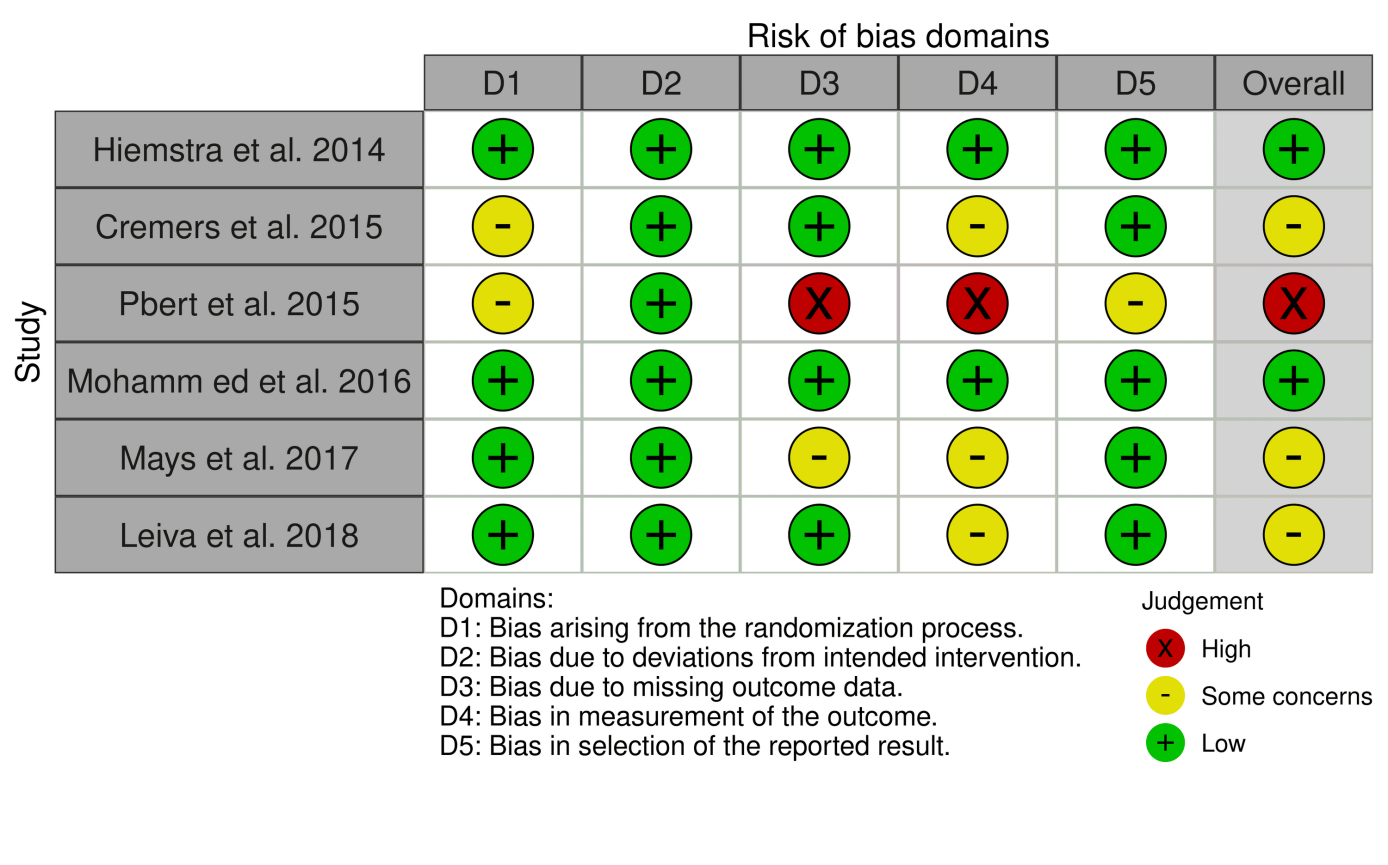
RoB 2 quality assessment of six RCT studies References: [6,3,17,13,19,11] RCT: randomized controlled trial

Figure 2 shows the quality assessment of six RCT trials, which were assessed using the RoB 2 quality assessment technique [20]. Due to a significant risk of bias, only one study was excluded from the meta-analysis [19].

Table 3 shows all double-armed meta-analyses of tobacco smoking prevention interventions. We found that there was no significant difference in abstinence rates between the intervention and control groups (RR 1 (95% CI (0.99, 1.02), P-value = 0.08). There was no significant heterogeneity detected (I^2^ = 0%, P-value = 0.79).

**Table 3:** Double-arm analysis of interventions for prevention of tobacco smoking outcomes Abstinence rate: [6,11,13] Smoking initiation: 6 months: [13,6] 12 months: [3,6] 24 months: [3,6] 36 months: [6,11] Smoking consumption: [11,13]

Outcomes	No. of studies	No. of total events in the intervention group	No. of total events in the control group	Risk ratio (95% CI)	P-value	Heterogeneity	
						I2	P-value
Abstinence rates	3	1591	1720	1 (0.99, 1.02)	0.80	0%	0.79
Smoking initiation after 6 months	2	22	59	0.38 (0.23, 0.61)	<0.001	0%	0.80
Smoking initiation after 12 months	2	16	21	0.69 (0.29, 1.32)	0.29	0%	0.51
Smoking initiation after 24 months	2	32	42	0.73 (0.46, 1.15)	0.17	0%	0.85
Smoking initiation after 36 months	2	175	207	-0.00 (-0.32, 0.03)	1	23%	0.26
Smoking consumption	2	158	217	0.76 (0.55, 1.05)	0.10	25%	0.25

After six months of intervention, there was a significant difference in the initiation of smoking, with the intervention group preferring the RR of 0.38 (95% CI (0.23, 0.61), P-value < 0.001). Low heterogeneity that was not significant was discovered (I^2 ^= 0%, P-value = 0.80). There was no significant difference between the intervention and control groups after 12, 24, and 36 months, with a RR of 0.69 (95% CI (0.29, 1.32), P-value = 0.29) for 12 months, 0.73 (95% CI (0.46, 1.15), P-value = 0.17) for 24 months, and -0.00 (95% CI (-0.32, 0.03), P-value = 1) for 36 months. For 12 months, I^2 ^= 0%, P-value = 0.51, for 24 months, I^2 ^= 0%, P-value = 0.85, and for 36 months, I^2 ^= 23%, P-value = 0.26, indicating a non-significant low heterogeneity.

Following the intervention, our meta-analysis showed a non-significant difference in smoking intake, with an RR of 0.76 (95% CI (0.55,1.05), P-value = 0.10). There was a non-significantly high level of heterogeneity (I^2^ = 25%, P-value = 0.25). A leave-one test was thus conducted. However, the heterogeneity remained high even after running the leave-one test.

Abstinence Rates

**Figure 3:**
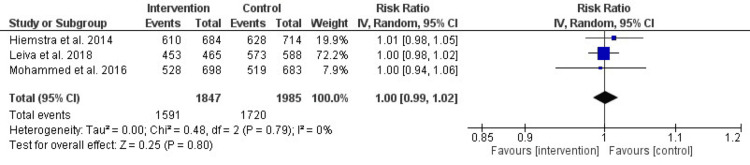
Forest plot of the effect of prevention interventions on the incidence of tobacco smoking References: [6,11,13]

Figure 3 presents the impact of prevention interventions on the incidence of tobacco smoking, visualized in a Forest plot. No significant differences in abstinence rates were observed between the intervention and control groups, with an RR of 1 (95% CI: 0.99-1.02, P = 0.08). Additionally, low heterogeneity was noted, with an I² value of 0% and a P-value of 0.79. These results suggest that the interventions did not have a significant effect on abstinence rates during the study period.

Smoking Initiation

**Figure 4:**
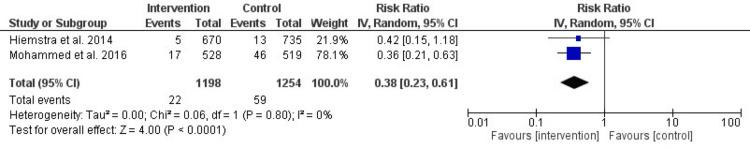
Forest plot of the effect of prevention interventions on smoking initiation after six-months of the intervention References: [6,13]

After six months: Figure 4 highlights the significant impact of prevention interventions on smoking initiation after six months. The intervention group showed a substantial reduction compared to the control group, with an RR of 0.38 (95% CI: 0.23-0.61, P < 0.001). Heterogeneity was low and non-significant (I² = 0%, P = 0.80). These results emphasize the intervention’s short-term efficacy in reducing smoking initiation.

**Figure 5:**
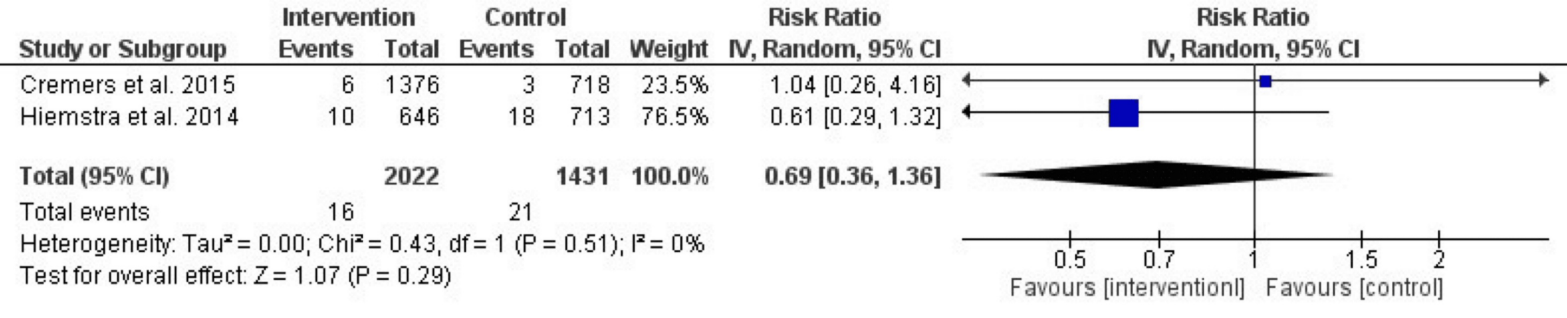
Forest plot of the effect of prevention interventions on smoking initiation after 12 months of the intervention References: [3,6]

After 12 months: Figure 5 shows no significant difference between the intervention and control groups. The RR was 0.69 (95% CI: 0.29-1.32, P = 0.29), with low and non-significant heterogeneity (I² = 0%, P = 0.51). This suggests that the intervention’s impact begins to diminish over time.

**Figure 6:**
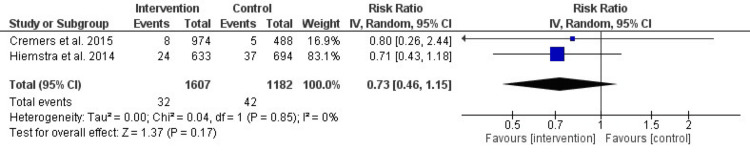
Forest plot of the effect of prevention interventions on smoking initiation after 24 months of the intervention References: [3,6]

After 24 months: The findings, illustrated in Figure 6, indicate no significant difference in smoking initiation rates after 24 months, with an RR of 0.73 (95% CI: 0.46-1.15, P = 0.17). Heterogeneity remained non-significant (I² = 0%, P = 0.85). This further supports the decline in the intervention’s long-term effectiveness.

**Figure 7:**
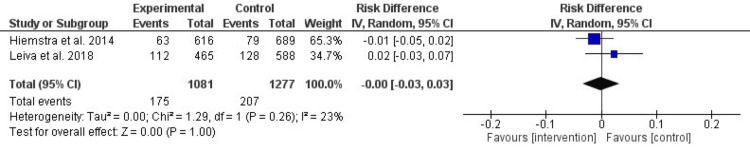
Forest plot of the effect of prevention interventions on smoking initiation after 36 months of the intervention References: [6,11]

After 36 months: As depicted in Figure 7, the intervention and control groups showed no significant differences in smoking initiation rates after 36 months. The RR was -0.00 (95% CI: -0.03 to 0.03, P = 1.0), and heterogeneity was low and non-significant (I² = 23%, P = 0.26). These results underscore the challenge of sustaining intervention effects over extended periods.

Smoking Consumption

**Figure 8:**
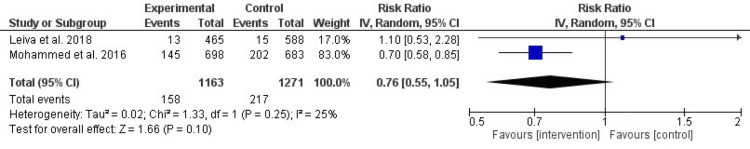
Forest plot of the effect of prevention interventions on smoking consumption after the intervention References: [11,13]

Figure 8 displays the Forest plot of smoking consumption rates following the intervention. While the intervention group showed a reduction in smoking consumption, the results were not statistically significant, with an RR of 0.76 (95% CI: 0.55-1.05, P = 0.10). Moderate heterogeneity was observed but remained non-significant (I² = 25%, P = 0.25). This suggests that while interventions may influence consumption patterns, the effects are inconsistent.

While some programs indicate beneficial changes in attitudes and engagement levels, the study emphasizes the varying effectiveness of smoking prevention techniques among children and adolescents. The effectiveness of smoking prevention techniques at this time was demonstrated by the study's finding that, after six months of intervention, there was a significant decrease in the initiation of smoking among adolescents. Enhancing the effectiveness of these programs and lowering teen smoking rates requires ongoing monitoring, removing implementation obstacles, and encouraging long-term educational support. Maintaining high standards of care and optimizing benefits requires striking a balance between these tactics and knowledge of any possible limitations.

Discussion

This systematic analysis focuses on the short-term effectiveness of school-based and culturally customized treatments in lowering smoking initiation rates among adolescents. Significant decreases were found at six months (RR = 0.38, 95% CI: 0.23-0.61, P < 0.001), according to the pooled analysis. These effects did not, however, last for extended follow-up times (12-36 months). In certain populations, like Saudi Arabia, subgroup analysis demonstrated the efficacy of peer-led, culturally tailored programs [6,13].

Comparison With Previous Studies

These results are consistent with research like Mohammed et al. (2016), which showed how important peer-led, culturally appropriate techniques are [13]. However, the findings are different from those of Cremers et al. (2015) and Hiemstra et al. (2014) which reported limited long-term impact of interventions [3,6]. This difference emphasizes the difficulty of maintaining behavioral changes over time, as well as the requirement for context-specific solutions [22-25].

Strengths and Limitations

The focus on RCTs and adherence to PRISMA standards are the review's strongest points [12]. However, disadvantages include the reliance on self-reported data, limited follow-up periods, and inconsistency in intervention implementation. It's possible that selection bias was introduced by excluding non-English research, which would have limited generalizability [5,10].

The results have practical implications as they highlight the value of culturally specific programs and brief, school-based interventions in lowering teen smoking initiation. Policymakers should prioritize putting these policies into action, especially in culturally diverse or high-risk communities. These programs can be made more effective by including community and family support [11,13,15].

Future Study Directions

To maintain long-term behavioral changes, future studies should look into multifaceted approaches that include digital tools, family engagement, and community-based efforts. Technologies that have the potential to increase adherence and engagement include virtual reality and web-based interventions [19,3]. Additionally, extended follow-up periods should be incorporated into studies to assess long-term results [22,25].

## Conclusions

The effectiveness of brief, non-pharmacological interventions - particularly those that are school-based and culturally specific - in reducing adolescent smoking initiation is demonstrated by this study. While significant reductions were observed at six months (RR = 0.38, 95% CI: 0.23-0.61, P < 0.001), these effects diminished over longer follow-up periods. To enhance long-term impact, future strategies should incorporate holistic approaches that engage families and communities, leverage digital technologies, and address challenges in implementation. Public health professionals and policymakers must prioritize culturally tailored interventions to increase reach and sustainability.

This review is limited by short follow-up periods, reliance on self-reported outcomes, and variability in intervention designs. Future research should explore innovative, multifaceted strategies with extended follow-up durations to sustain behavioral changes. Despite these limitations, the findings emphasize the importance of equipping schools and communities with effective resources to protect future generations from the harmful consequences of smoking and contribute to global efforts to curb the tobacco epidemic.
